# BuyangHuanwu Decoction alleviates Endothelial Cell Apoptosis and Coronary Microvascular Dysfunction via Regulation of the MAPKK4/p38 Signaling Axis

**DOI:** 10.7150/ijms.98183

**Published:** 2024-09-23

**Authors:** Xing Chang, Dan Wu, Xin Gao, Jianguo Lin, Ying Tan, Junyan Wang, Hang Zhu, Hao Zhou

**Affiliations:** 1 Guang'anmen Hospital, China Academy of Chinese Medical Sciences, Beijing, 100053, China.; 2 Division of Vascular Surgery, the First Affiliated Hospital, Sun Yat-sen University, Guangzhou 510800, China; National-Guangdong Joint Engineering Laboratory for Diagnosis and Treatment of Vascular Disease, First Affiliated Hospital, Sun Yat-sen University, Guangzhou, 510080, China.; 3 Outpatient Department of the Sixth Medical Center of the PLA General Hospital, China.; 4 The First Affliated Hospital of Zhejiang Chinese Medical University, Hangzhou, China.; 5 Beijing University of Chinese Medicine, Beijing, 100028, China.; 6 School of Pharmaceutical Sciences, Guangzhou University of Chinese Medicine, Guangzhou, Guangdong, 510006, China; 7 Senior Department of Cardiology, The Sixth Medical Center of People's Liberation Army General Hospital, Beijing, China.

**Keywords:** MAPKK4, P38, Coronary microvascular injury, Buyang Huanwu decoction, Cell apoptosis, Inflammatory injury

## Abstract

MAPKK4 has been implicated in the pathological mechanisms underlying myocardial and vascular injury, specifically influencing endothelial cell damage and programmed cell death via subcellular pathways. Nevertheless, the regulatory role of MAPKK4 in coronary microvascular injury following myocardial infarction remains unconfirmed, and the exploration of targeted mitochondrial protective therapeutic agents remains unaddressed. In light of this gap, we established a MAPKK4 gene-modified mouse model of ischemia-reperfusion injury and employed Buyang Huanwu decoction (BYHW), a traditional cardiovascular therapeutic formula, to assess its efficacy in treating coronary microvascular injury post-ischemia-reperfusion. The study aimed to elucidate the mechanism by which BYHW mitigates coronary microvascular injury induced by ischemia-reperfusion through the attenuation of endothelial cell apoptosis. Experimental outcomes revealed that high-dose BYHW significantly ameliorated coronary microvascular injury post-ischemia-reperfusion, restoring the structural integrity of the coronary microvasculature and reducing inflammation and oxidative stress. Contrarily, in transgenic mice overexpressing MAPKK4, BYHW intervention failed to attenuate microvascular inflammation and oxidative stress. To further investigate, we simulated hypoxia/reoxygenation injury in vascular endothelial cells using a MAPKK4-related cellular gene modification model. The results indicated that BYHW attenuates inflammatory damage and enhances the viability of vascular endothelial cells following hypoxic stress, inhibiting apoptosis via the mitochondrial pathway. However, overexpression of MAPKK4/p38 negated the therapeutic effects of BYHW, showing no impact on endothelial cell apoptosis and oxidative stress under hypoxic conditions. Molecular interaction studies confirmed that the active components of BYHW, Astragaloside IV and Ligustrazine, interact with the MAPKK4/P38 axis. *In vitro* experiments further suggested that the interaction between MAPKK4 and P38 play a crucial role in the ability of BYHW to inhibit apoptosis in coronary microvascular endothelial cells. Therapeutically, MAPKK4 may potentiate the apoptotic pathway in microvascular endothelial cells by modulating downstream P38 expression and phosphorylation, thereby exacerbating ischemia-reperfusion-induced coronary microvascular endothelial injury. From an *in vivo* perspective, the transgenic overexpression of MAPKK4 and P38 inhibited the microvascular protective effects of BYHW. These findings collectively underscore the significance of the MAPKK4-P38 axis in the protection of coronary microvascular endothelial cells.

## Introduction

The pathological and physiological foundations of coronary microcirculation injury are intricate, marked by the interplay of multiple processes, including ischemia-induced injury and hypoxia stress response 1, 2. Early research primarily supported the hypothesis that no-reflow results from microvascular obstruction, with some studies also highlighting irreversible microvascular damage and subsequent myocardial hemorrhage as critical contributors to no-reflow 3. Currently, various studies have identified key pathophysiological factors underlying coronary microvascular injury, including oxidative stress, mitochondrial dysfunction, inflammatory cascade reactions, programmed cell death, endoplasmic reticulum stress, and calcium overload, among other mechanisms 4. Substantial evidence has underscored the pivotal role of mitochondrial pathway-induced apoptosis and oxidative stress in maintaining cellular homeostasis.

Our previous research has elucidated the regulatory mechanisms of mitochondrial energy metabolism disorders, programmed cell death via mitochondrial pathways, and mitochondrial oxidative stress in the context of coronary microvascular injury 5, 6. We have also validated the interaction mechanisms between downstream targets and protein sites within the mitochondrial quality control regulatory pathway 7, 8. Additionally, further investigations into the pathological changes associated with ischemic myocardial injury before, during, and after coronary microvascular injury revealed a close association between the MAPPK4-P38 signaling pathway and microcirculation injury 7, 9.

Despite numerous studies suggesting the targeted regulatory effects of this pathway on endothelial and myocardial cells, the development of targeted therapeutic drugs and elucidation of myocardial/endothelial protective mechanisms specific to this pathway remain incomplete 10. Coronary microvascular endothelial cells play a crucial role in endothelial injury post-ischemia-reperfusion, and the P38 mitogen-activated protein kinase (MAPK) is among the key signaling pathways facilitating endothelial cell adaptation to ischemia/hypoxia and nutrient deprivation 11, 12. While p38-MAPK is known to regulate ATPase synthesis and oxidative phosphorylation metabolism during stress injury, research into its targeted therapeutic applications is ongoing. Multiple studies have affirmed the regulatory role of the mitogen-activated protein kinase (MAPK) signaling cascade, including MAPKKK10, MAPKK4, and p38 MAPK, in myocardial and endothelial stress injury 13. However, the development of targeted regulatory drugs remains in the research phase. Buyang Huanwu decoction (BYHW) is a traditional formula predominantly composed of Huangqi (Astragalus membranaceus), Danggui (Angelica sinensis), Chishao, Dilong, Chuanxiong, and Honghua, among others, and is a well-established therapeutic agent for cardiovascular diseases 14-16. Flavonoids present in Astragalus membranaceus, such as Astragalus polysaccharides and Astragaloside IV, exhibit significant anti-inflammatory, antioxidant, and metabolic regulatory properties, contributing to the protection of cardiovascular and myocardial cells 17, 18. Similarly, the constituents of Angelica sinensis, including ferulic acid and Angelica sinensis polysaccharides, provide antioxidant protection and inhibit myocardial apoptosis, thereby preserving myocardial and vascular endothelial function 19, 20. The combination of Ligusticum chuanxiong and Angelica sinensis is particularly effective in exerting anti-inflammatory and analgesic effects, primarily by inhibiting inflammatory mediators such as TNF-α and IL-6. Ferulic acid and caffeic acid, common components of both Angelica sinensis and Ligusticum chuanxiong, work synergistically to deliver potent antioxidant effects 21, 22. Additionally, this combination demonstrates anticoagulant properties, lowers blood lipid levels, and inhibits platelet activation. The multiple active ingredients in BYHW collectively offer robust myocardial and vascular protection, alongside anti-inflammatory, antioxidative, and anti-apoptotic effects. Endothelial cell apoptosis, a form of programmed cell death, is influenced by a range of factors including inflammatory damage, oxidative stress, calcium homeostasis disruption, and various stress stimuli targeting key subcellular organelles such as mitochondria and the endoplasmic reticulum 23, 24. The apoptotic pathway is tightly regulated by upstream proteins in endothelial cells, particularly in the context of cellular inflammation 25. The caspase family, as markers of apoptosis, can be activated by inducing factors that destabilize cellular homeostasis 26, 27. Given the regulatory influence of the MAPKK4-p38 axis on myocardial and vascular endothelial cells and the targeted therapeutic potential of BYHW, we undertook a series of studies to investigate the interaction mechanisms in both animal and cellular models. These studies, conducted through both *in vitro* and *in vivo* experiments, validated the protective effects of BYHW and further elucidated the mechanism by which BYHW ameliorates coronary microvascular injury post-ischemia-reperfusion by modulating cell apoptosis through the MAPKK4-p38 pathway.

## Materials and Methods

### Ethical statement

This study was performed in strict accordance with the recommendations in the Guide for the Care and Use of Laboratory Animals at the School of Pharmaceutical Sciences, Guangzhou University of Chinese Medicine. Animal procedures were performed according to protocols approved by the Animal Ethics Committee at Guangzhou University of Chinese Medicine (No. 20211207002).

### Experimental animals

MAPKK4^CKO/TG^ and p38^TG^ mice were generated based on previous studies 28-30. This study was approved by the Ethics Center of Guangzhou university of Traditional chinese medicine in accordance with the ARRIVE Guidelines for Animal Research. Adult male C57BL/6 mice, aged 7-8 weeks, were homogenously housed at the Animal Experiment Center of Guangzhou university of Traditional chinese medicine. The laboratory maintained a temperature of 23-25 C, followed a 12h light/dark cycle, sustained a humidity level of 55% ± 5%, and supplied approved standard food and free access to water for a minimum of 7 days.

### Animals model

Establish a coronary microvascular injury model through myocardial ischemia-reperfusion model 7, 8. C57BL/6 mice were anesthetized by intraperitoneal injection of 50mg/kg pentobarbital. After confirming the anesthesia status, use hair removal cream for hair removal. The temperature range of the small constant temperature operating table is 35-37 ℃, and a cardiac monitor is installed. The prepared skin is locally disinfected, the intercostal skin is cut open, the pericardium is opened, the heart is pushed out of the body, and the left anterior descending coronary artery is quickly ligated after exposure. When the bottom heart turns white, quickly close the chest and squeeze to prevent pneumothorax, and suture the muscles and skin layer by layer 31. The surgery resulted in myocardial ischemia of the left ventricular anterior wall. After 30-45 minutes of ischemia, perform a second thoracotomy to ligate and release the ligature, restoring blood flow to the left outer branch of the coronary artery and myocardial tissue. Close the chest and compress to prevent pneumothorax, and suture the muscles and skin layer by layer. Similarly, the mice in the sham surgery group received anesthesia, thoracotomy, and thread insertion without causing myocardial ischemia 32, 33.

### Cell culture

In order to conduct *in vitro* modeling of coronary microvascular cells, oxygen glucose deprivation was used to simulate ischemia, and oxygen glucose supply was restored to simulate reperfusion 34. Use a pre saturated simulated ischemic solution of 95% N2+5% CO2 instead of culture medium, and incubate cells in a cell culture box containing 95% N2 followed by incubation with DMED simulated reperfusion solution containing 10% fetal bovine serum instead of simulated ischemic solution for 3 hours 35. All groups underwent the aforementioned ischemia-reperfusion procedure. Cells were cultured in DMEM supplemented with glutamine, 10% fetal bovine serum, and 100 μ g/mL penicillin/streptomycin. Cells were transfected with MAPKK4/P38 adenovirus to obtain overexpression or knockout gene modification treatment 32, 36.

### Enzyme linked immunosorbent assay

The enzyme-linked immunosorbent assay (ELISA) kits for creatine kinase (CK-MB), troponin T, and lactate dehydrogenase (LDH) were purchased from Wuhan Feien Biotechnology Co., Ltd. (Wuhan, China) and measured according to the manufacturer's instructions 37, 38.

### Transmission electron microscopy detection

Determine the sampling site of fresh coronary microvascular tissue using transmission electron microscopy to minimize mechanical damage. Prior to sampling, a culture dish containing a fixed solution for electron microscopy was prepared in advance. Immediately place the tissue into a culture dish and cut it into small pieces to prevent it from settling in the fixed solution 39. Then, add these small woven blocks with fresh electron microscope fixative. Then rinse the tissue three times with 0.1M phosphate buffer (pH 7.4) for 15 minutes each time. Copper mesh slices were stained and dried overnight in copper cages in the room. Analyze images under transmission electron microscopy 40.

### Immunofluorescence in paraffin sections

Paraffin sections of myocardial tissue were deparaffinized, and the tissue sections were placed in EDTA antigen repair buffer (pH 8.0) and removed in a microwave oven. After the remaining part is slightly dried, use a chemical pen to draw circles around the tissue (to prevent antibody loss) 41, dry, rinse with phosphate buffered saline (PBS), block in bovine serum albumin, and then seal. Then cultivate the glass slide with primary and secondary antibodies in sequence, and keep it away from light at room temperature for 50 minutes 42. After retraining with DAPI, the nuclei and sections were dried and sealed with anti-fluorescence quenching slides. Observe under a fluorescence electron microscope 43.

### Mitochondrial respiratory chain function

Each group of myocardial cells was used for ELISA detection. Mitochondrial respiratory complex I (ab109721, Abcam, MA, USA), mitochondrial respiratory complex II (ab109908, Abcam), and mitochondrial respiratory complex III (A089-3-1, manufactured by Nanjing Jiancheng) were evaluated. Wash the cells with PBS and mix. Grind or repeatedly freeze thaw after ultrasonic treatment, centrifuge the homogenate at 5000g and stir for 5-10 minutes, and use the supernatant for detection 44.

### Real-time quantitative PCR

Extract 10mg of total RNA from heart tissue using TRIzol reagent (Invitrogen), followed by chloroform purification and isopropanol precipitation. Use NanoDrop instrument (Thermo Fisher Scientific) to detect RNA concentration 45. Reverse transcribe RNA using PrimeScriptTM RT kit and amplify the obtained cDNA using a rapid real-time PCR instrument (ABI-7900-384, Applied Biosystems) equipped with TB Green Premix Ex TaqTM II (RR820A, Takara, Japan). QPCR was used to amplify the cytochrome c oxidase subunit I (CO1) gene and NDUFV1 nDNA gene of mtDNA 46, 47. The reaction was initiated at 94 ° C for 10 minutes, followed by 40 cycles at 94 ° C for 10 seconds, 60 ° C for 30 seconds, and 94 ° C for 10 seconds. All reactions were repeated. Use SDS 1.9.1 software (Applied Biosystems) to analyze amplification curves and determine the relative ratio of mtDNA: nDNA in each sample based on previous studies using these curves 48, 49.

### Statistical analysis

Use GraphPad Prism 9.0 statistical software to analyze the results. The data is displayed as mean ± standard error of mean (SEM). For the comparison between the two groups, parametric Student t-test or nonparametric Mann Whitney test was used. For comparisons between two or more groups, use parametric one-way ANOVA test, followed by Bonferroni test. The histopathological parameters of the tissue were statistically analyzed using chi square test. P<0.05 is considered significant.

## Results

### Buyang Huanwu decoction improves coronary microvascular injury by regulating inflammation and cell apoptosis

**Figure 1:**
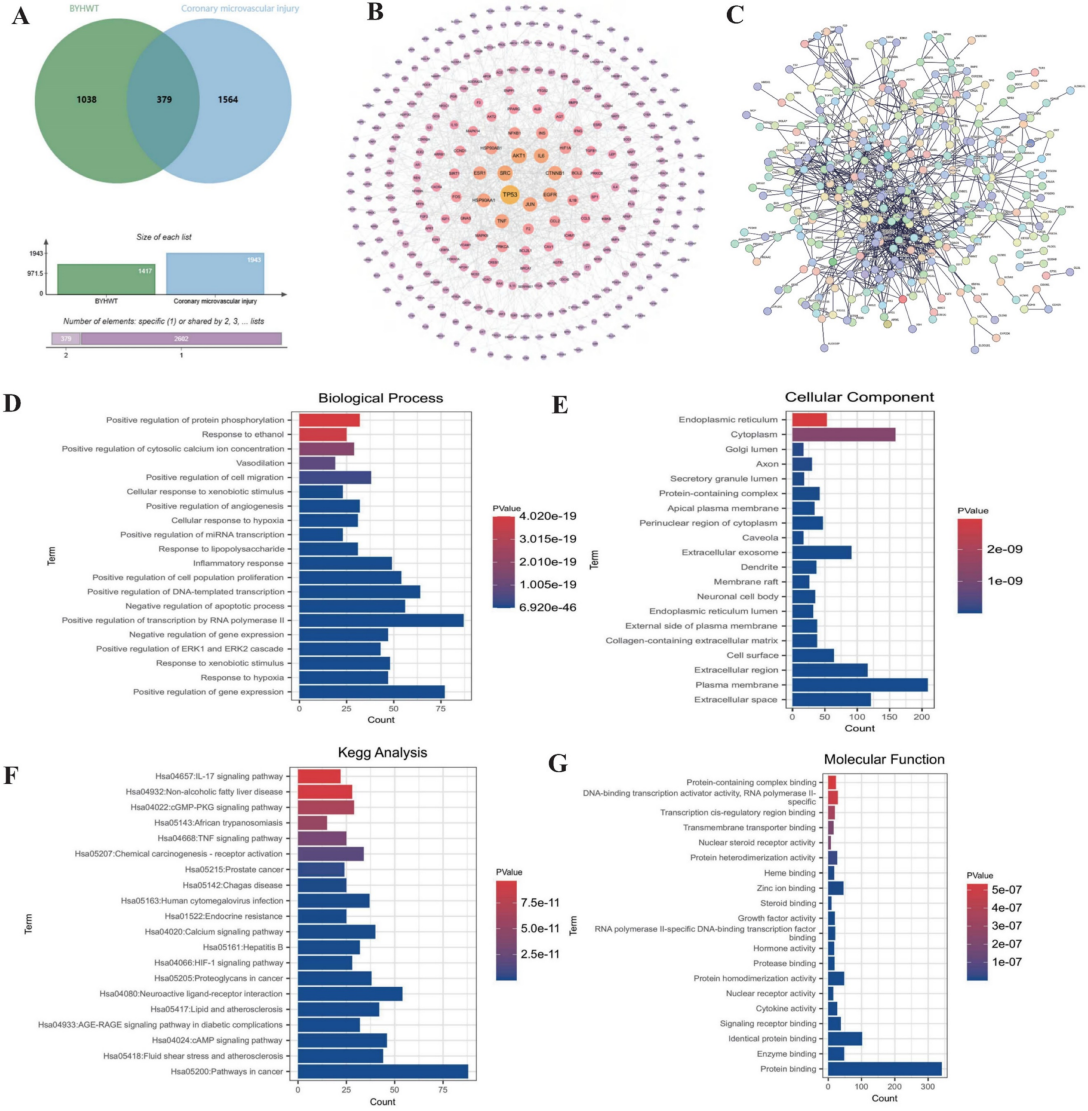
** Network pharmacology analysis of the mechanism of action of Buyang Huanwu decoction in treating coronary microvascular injury after ischemia-reperfusion injury. (A)** Wayne diagram analysis of the intersection targets between BYHW and coronary microvascular injury; **(B-C)** PPI network analysis of the targets and signaling pathways of BYHW in treating coronary microvascular injury; **(D-G)** KEGG and GO enrichment analysis of differentially expressed genes and the mechanism of action of BYHW.

**Figure 2:**
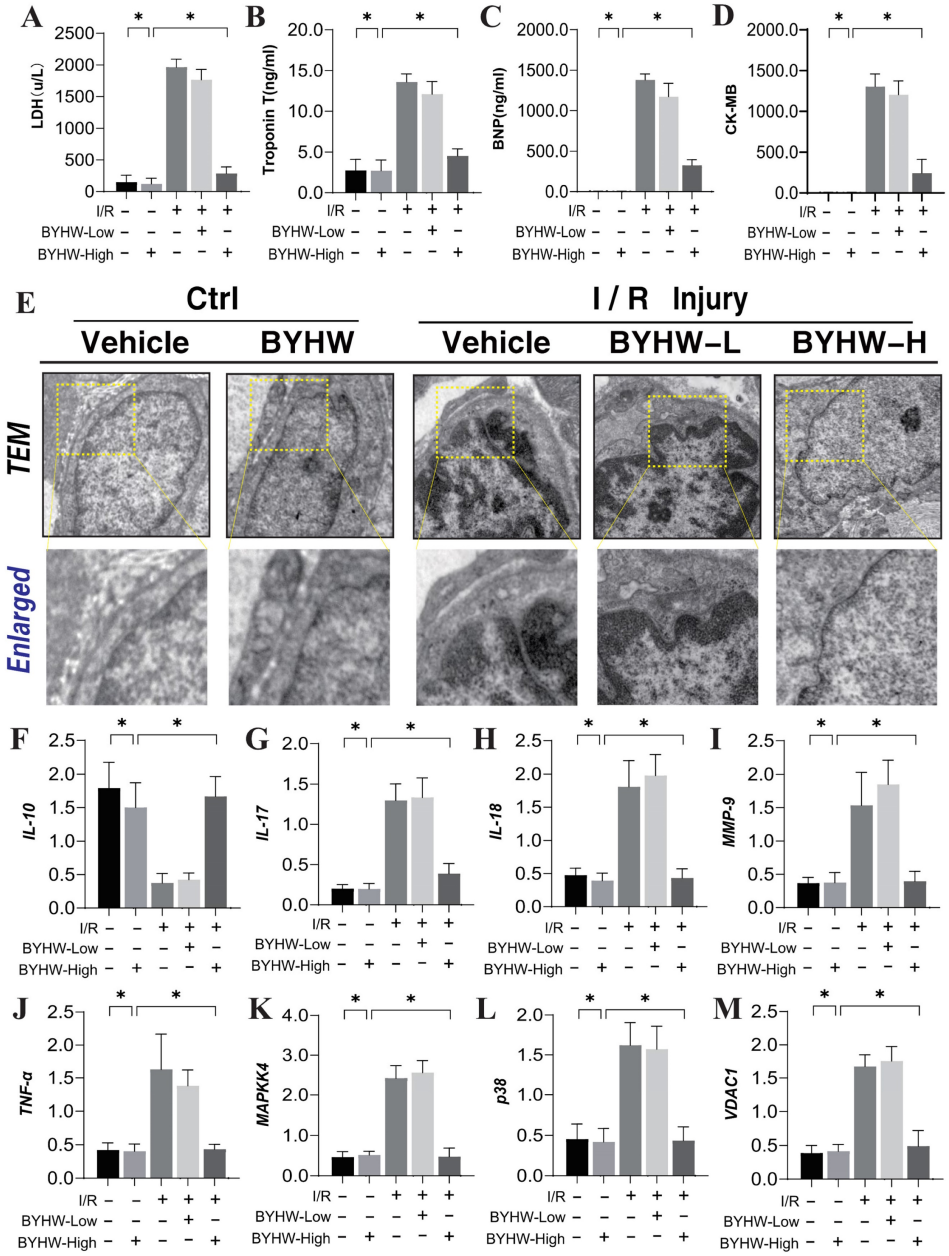
** Buyang Huanwu decoction improves coronary microvascular injury after IR by regulating Caspase mediated cell apoptosis through MAPK44. (A)** LDH expression level; **(B)** Troponon T expression level; **(C)** BNP expression level; **(D)** CK-MB expression level; **(E)** Representative TEM images of heart tissues from control and SCM mice. White arrows indicate disorganized myofibrils and tortuous Z discs while yellow arrows indicate swollen mitochondria. Scale bar, 2 μm **(F)** IL-10 expression level; **(G)** IL-17 expression level; **(H)** IL-18 expression level; **(I)** MMP-9 expression level; **(J)** TNF - α expression level; **(K)** MAPKK4 expression level; **(L)** P38 expression level; **(M)** VDAC1 expression level; Data are shown as mean ± SEM (n=10 mice per group or ten independent cell isolations per group). *p<0.05.

To investigate the mechanisms underlying coronary microvascular injury following ischemia-reperfusion (IR), we assessed the therapeutic effects of Buyang Huanwu decoction (BYHW) on this condition, using network pharmacology. Through this analysis, we identified key drug targets and mechanisms, revealing that protective effects of BYHW on coronary microvascular injury are primarily associated with the mitigation of inflammatory injury, endoplasmic reticulum stress, protein homeostasis disruption, transmembrane transport dysfunction, and impaired energy metabolism (Figure 1A-G). To further validate the mechanisms by which BYHW ameliorates coronary microvascular injury post-IR, we established a mouse model of coronary microvascular injury induced by IR and administered BYHW without a dose gradient for intervention. Transmission electron microscopy results demonstrated classic indicators of microvascular damage in the modeled mice, such as structural compromise, stenosis, and disrupted blood flow (Figure 2E). Importantly, BYHW did not affect the structural integrity or function of coronary microvasculature in control group mice. However, high-dose BYHW markedly improved microvascular damage, preserving the structural integrity of the microvessels (Figure 2E), whereas low-dose BYHW failed to provide substantial improvement in microvascular structural damage (Figure 2E).

Further biochemical assays measuring LDH, Troponin T (Trop T), BNP, and CK-MB indicated significant elevations of these markers following IR modeling, reflective of severe myocardial and microvascular damage (Figure 2A-D). High-dose BYHW significantly reversed these effects, reducing the expression levels of LDH, Trop T, BNP, and CK-MB, thereby mitigating microvascular damage (Figure 2A-D). In contrast, low-dose BYHW did not effectively reverse these elevations (Figure 2A-D). Additionally, post-IR, a marked reduction in the anti-inflammatory factor IL-10 was observed in microvascular tissues, accompanied by significant increases in pro-inflammatory factors IL-17, IL-18, and MMP-9, indicative of an inflammatory storm (Figure 2F-J). High-dose BYHW was able to counteract this inflammatory surge, restoring IL-10 levels and suppressing the upregulation of IL-17, IL-18, and MMP-9, thereby alleviating the microvascular inflammatory response post-IR (Figure 2F-J). Conversely, low-dose BYHW was ineffective in ameliorating the inflammatory damage (Figure 2F-J). Mechanistic studies revealed a significant upregulation of MAPK44/P38 and VDAC1 expression following IR, suggesting that MAPK44/P38-mediated overexpression of VDAC1 may contribute to energy metabolism dysfunction and disruptions in membrane permeability transition pores, further exacerbating microvascular inflammatory storms and structural damage (Figure 2K-L). Notably, high-dose BYHW effectively inhibited the expression of MAPK44/P38 and VDAC1 in microvessels post-IR, whereas low-dose BYHW did not reverse the overexpression of MAPK44/P38 (Figure 2K-L). These findings suggest that the MAPK44/P38 axis is a critical regulatory mechanism in the therapeutic effects of BYHW on microvascular injury post-IR.

### Buyang Huanwu decoction improves coronary microvascular injury after IR by regulating Caspase mediated cell apoptosis through MAPK44

**Figure 3:**
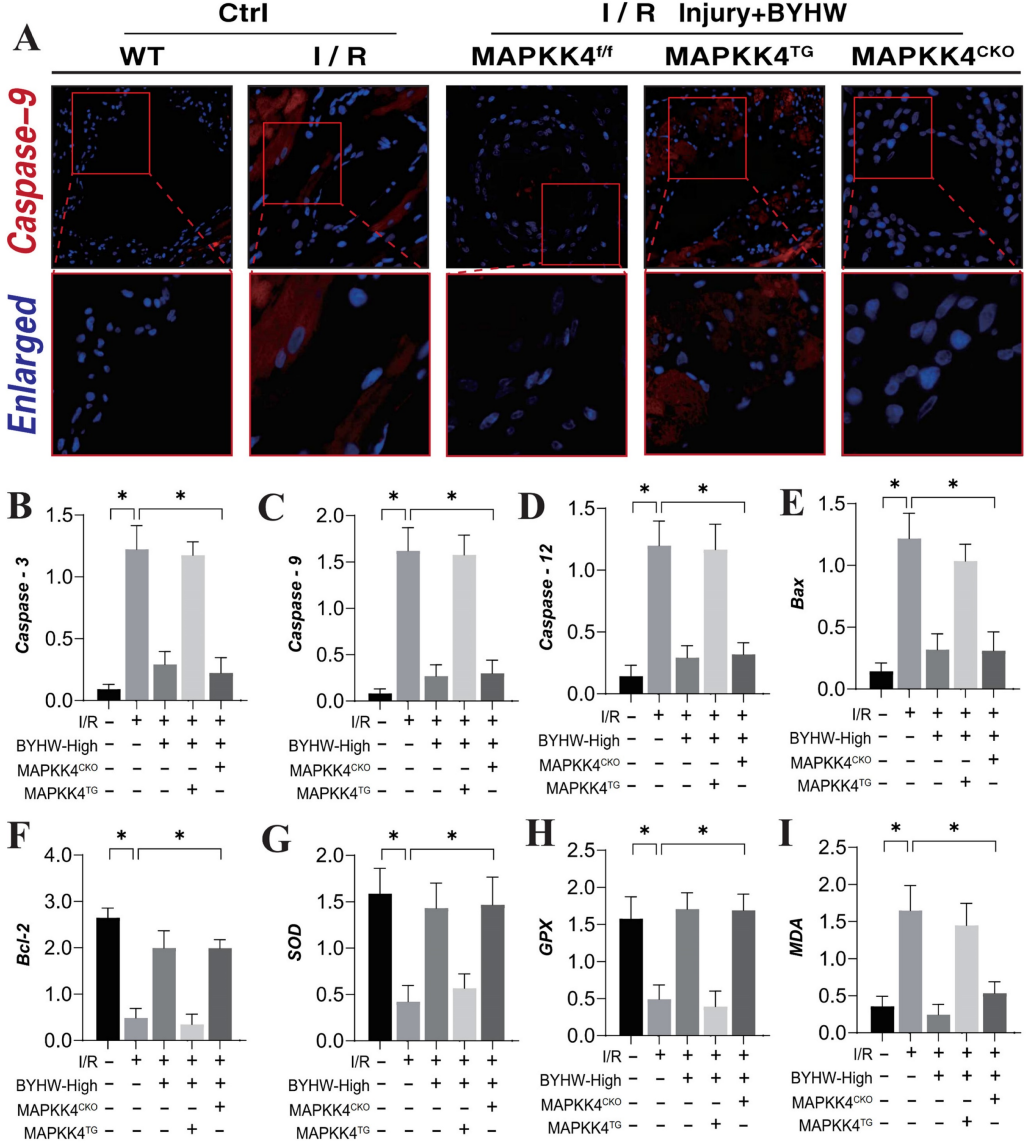
** Buyang Huanwu decoction improves coronary microvascular injury after IR by regulating energy metabolism and cell apoptosis through MAPK44-P38. (A)** Organize immunofluorescence detection of Caspase-9 expression levels; **(B)** Caspase-3 transcription level; **(C)** Caspase-9 transcription level; **(D)** Caspase-12 transcription level; **(E)** Bax expression level; **(F)** Bcl-2 expression level; **(G)** SOD expression level; **(H)** GPX expression level; **(I)** MDA expression level; Data are shown as mean ± SEM (n= ten independent cell isolations per group). *p<0.05.

Our previous studies have established that the Caspase-mediated mitochondrial pathway is a critical pathological regulator of apoptosis and necroptosis in coronary microvascular endothelial cells during coronary microvascular injury 50-52. This study further corroborates the mechanism underlying apoptotic damage, demonstrating a significant increase in Caspase-9 expression within microvascular tissue following ischemia-reperfusion (IR) modeling. Notably, high-dose Buyang Huanwu decoction (BYHW) treatment effectively reversed the elevated fluorescence expression of Caspase-9 (Figure 3A). Interestingly, the introduction of MAPKK4 transgenic treatment attenuated the regulatory effect of BYHW on Caspase-9 expression levels. In contrast, cardiac-specific knockout of MAPKK4 did not diminish the therapeutic efficacy of BYHW (Figure 3A).

Further analysis of serum levels of Caspase-3, Caspase-9, and Caspase-12 supported these findings, showing consistency with the results observed in microvascular tissue fluorescence detection (Figure 3B-D). Moreover, the assessment of Bax/Bcl-2 ratios and oxidative stress-related markers indicated that IR modeling led to an increase in Bax and the oxidative stress marker MDA, alongside a reduction in Bcl-2 and antioxidant enzymes SOD and GPX (Figure 3E-I). BYHW effectively reversed these changes, inhibiting the activation of apoptotic pathways and reducing oxidative stress damage (Figure 3E-I). These results suggest that BYHW ameliorates microvascular damage post-IR by modulating apoptosis through the mitochondrial pathway and mitigating oxidative stress. It is noteworthy that the therapeutic benefits of BYHW were negated by MAPKK4 gene knockout, while MAPKK4 transgenic treatment did not reverse the protective effects of BYHW.

### Buyang Huanwu decoction improves coronary microvascular injury after IR by regulating energy metabolism and cell apoptosis through MAPK44-P38

**Figure 4:**
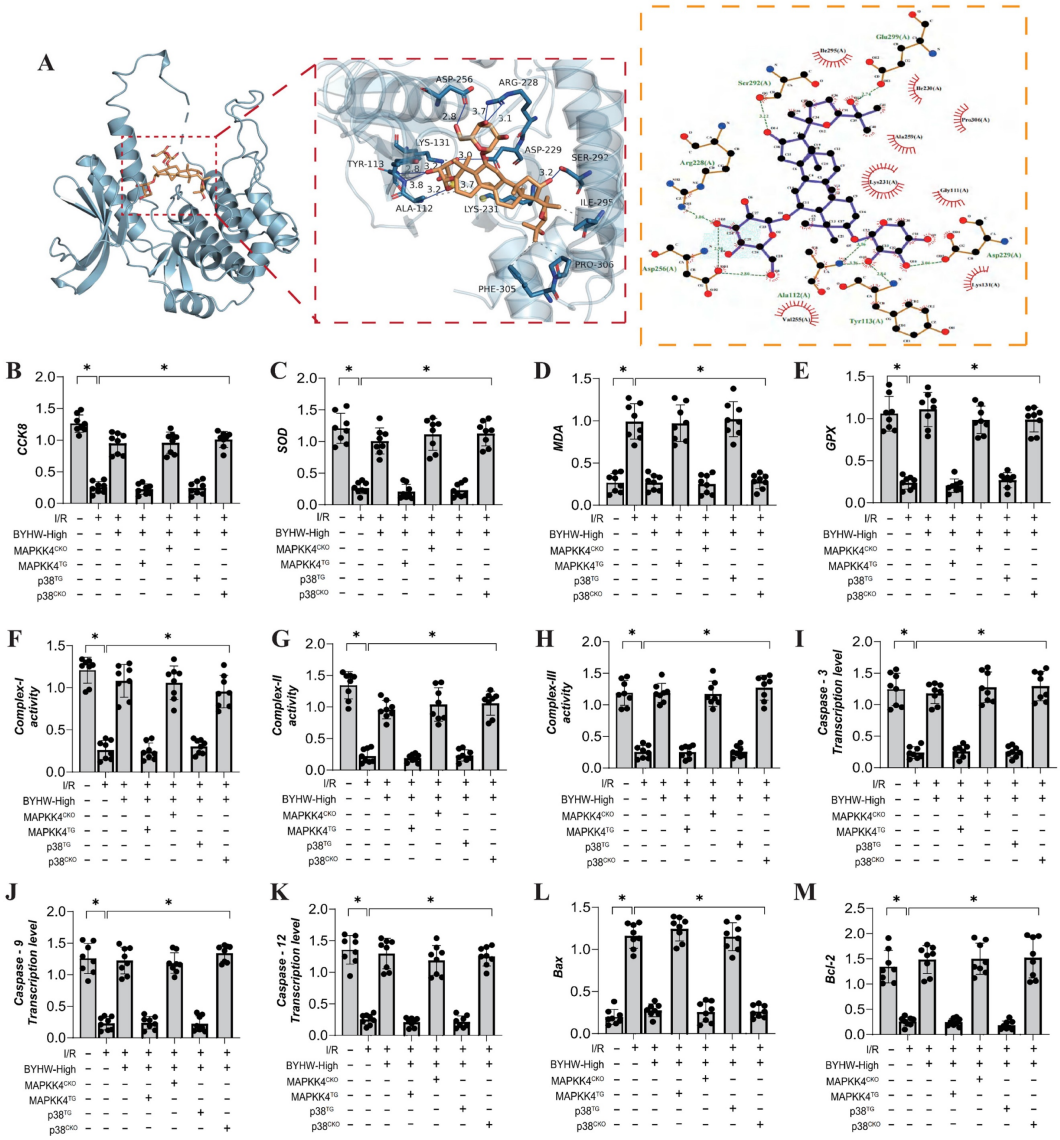
** Buyang Huanwu decoction improves coronary microvascular injury by inhibiting cell apoptosis through MAPPK4-P38 interaction. (A)** Molecular docking correlation prediction between MAPKK4 and Astragaloside IV; **(B)** CCK8 detects the activity of coronary microvascular endothelial cells; **(C)** SOD expression level; **(D)** MDA expression level; **(E)** GPX expression level; **(F)** Complex-I activity; **(G)** Complex II activity; **(H)** Complex III activity; **(I)** Caspase-3 transcription level; **(J)** Caspase-9 transcription level; **(K)** Caspase-12 transcription level; **(L)** Bax expression level; **(M)** Bcl-2 expression level; Data are shown as mean ± SEM (n= ten independent cell isolations per group). *p<0.05.

To further elucidate the mechanism of action of Buyang Huanwu decoction (BYHW), we conducted molecular docking experiments to simulate the interaction between MAPKK4 and Astragaloside IV, one of the active ingredients in BYHW. The results demonstrated a high affinity between MAPKK4 and Astragaloside IV, reinforcing the hypothesis that MAPKK4 is a critical target for the microvascular therapeutic effects of BYHW (Figure 4A). To verify the therapeutic effects of BYHW at a cellular level, we utilized gene modification technology to create models of MAPK44 and P38 knockdown/overexpression in microvascular endothelial cells, followed by hypoxia/reoxygenation experiments to simulate ischemia/reperfusion (IR) injury. The experimental results indicated a marked reduction in coronary microvascular endothelial cell activity post-modeling, which was accompanied by a decrease in the expression levels of antioxidant enzymes (SOD, MDA, GPX) and a suppression of mitochondrial respiratory chain complexes (Complex I/II/III) (Figure 4B-H). BYHW treatment effectively reversed these effects, enhancing the expression of SOD, MDA, and GPX, and upregulating the expression of mitochondrial respiratory chain complexes (Complex I/II/III) (Figure 4B-H).

Further results indicated that BYHW also inhibited the expression levels of apoptosis-related proteins Caspase-3, Caspase-9, Caspase-12, and Bax, while increasing the expression of the anti-apoptotic protein Bcl-2 (Figure 4I-M). Notably, transgenic treatments with MAPKK4 and P38 counteracted these beneficial effects of BYHW, whereas overexpression of MAPKK4/P38 did not diminish the protective effects of BYHW (Figure 4B-M). These experimental results confirm that BYHW enhances mitochondrial energy metabolism, mitigates oxidative stress, and protects against microvascular endothelial cell apoptosis via the MAPKK4/P38 pathway. This, in turn, boosts endothelial cell activity and preserves endothelial function. However, the precise regulatory mechanisms and crosstalk between BYHW and the MAPKK4-P38 signaling axis require further investigation.

### Buyang Huanwu decoction improves coronary microvascular injury by inhibiting cell apoptosis through MAPPK4-P38 interaction

**Figure 5:**
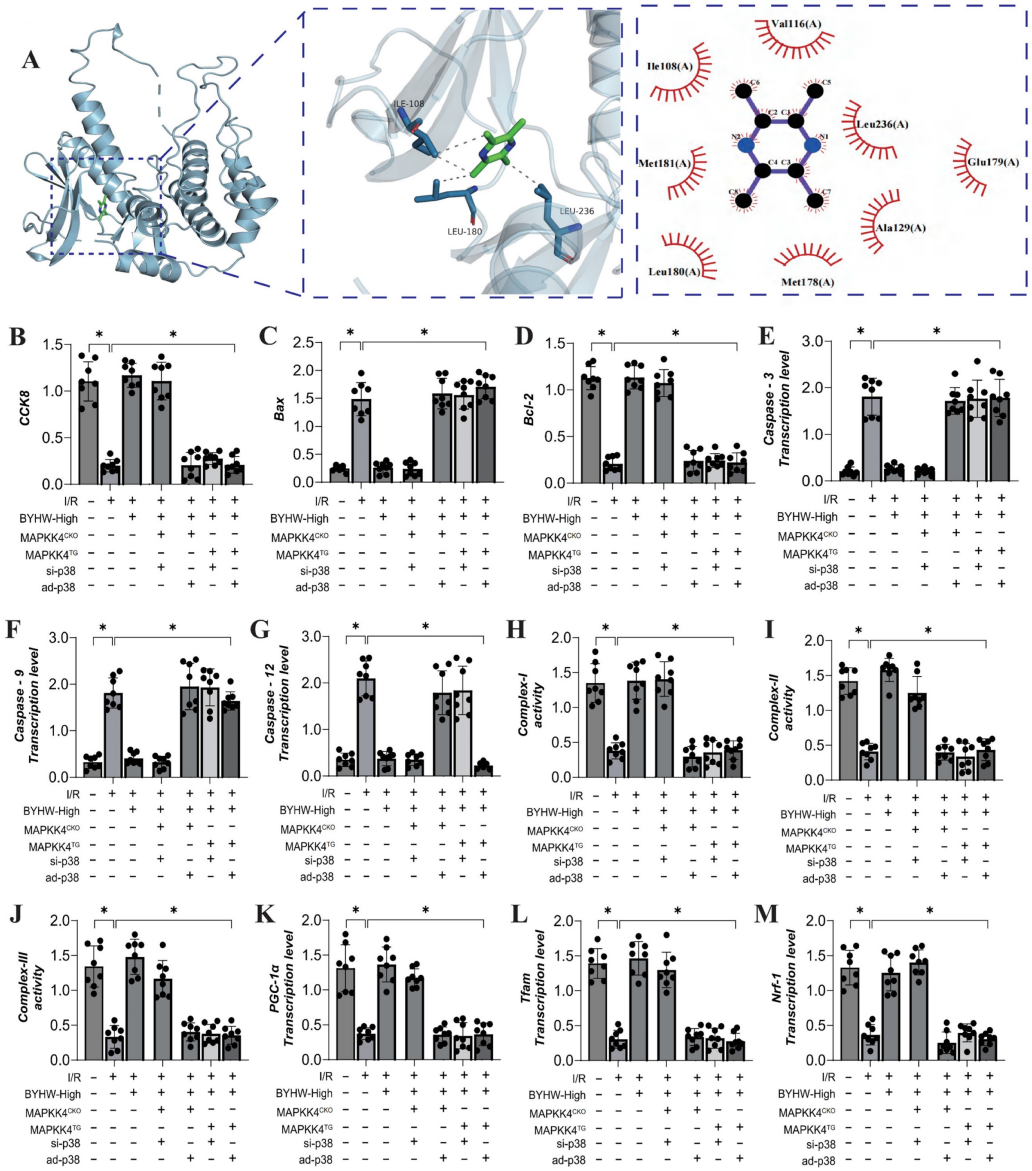
** Buyang Huanwu decoction improves coronary microvascular injury by inhibiting cell apoptosis through MAPPK4-P38 interaction. (A)** Molecular docking correlation prediction between MAPKK4 and ligustrazine; **(B)** CCK8 detects the activity of coronary microvascular endothelial cells; **(C)** Bax expression level; **(D)** Bcl-2 expression level; **(E)** Caspase-3 transcription level; **(F)** Caspase-9 transcription level; **(G)** Caspase-12 transcription level; **(H)** Complex-I activity; **(I)** Complex II activity; **(J)** Complex III activity; **(K)** PGC1- α transcription level; **(L)** Tfam transcription level; **(M)** Nrf-1 transcription level; Data are shown as mean ± SEM (n= ten independent cell isolations per group). *p<0.05.

We further investigated the interaction between MAPKK4 and the active ingredient ligustrazine in Buyang Huanwu decoction (BYHW) using molecular docking experiments. The results demonstrated a strong correlation between MAPKK4 and ligustrazine, suggesting a potential mechanism through which BYHW mitigates microvascular injury via MAPKK4 (Figure 5A). Additional experimental findings indicate that BYHW enhances mitochondrial energy metabolism, inhibits apoptosis via the mitochondrial pathway, and promotes mitochondrial biosynthesis (Figure 5B-M). However, treatments with MAPKK4 transgenic (MAPKK4^TG^) and MAPKK4^TG^/ad-p38 models blocked regulatory effects of BYHW on mitochondrial biosynthesis, mitochondrial pathway-induced apoptosis, and mitochondrial respiratory chain function (Figure 4B-M). In contrast, treatments with MAPKK4 conditional knockout (MAPKK4^CKO^) and MAPKK4^CKO^/s-p38 did not negate the protective effects of BYHW (Figure 4B-M).

These results further suggest that protective effects of BYHW on coronary microvascular endothelial cells may be mediated through the MAPKK4-P38 interaction pathway, consistent with our previous findings. Our earlier research also highlighted the crucial role of mitochondrial biosynthesis in the context of ischemia-reperfusion injury 53, 54. Mitochondrial biosynthesis is essential for constructing and modifying cellular structures, regulating reaction conditions, and optimizing cellular functions 55. The mitochondrial biosynthesis process is regulated by a variety of factors, including transcription factors, RNA stabilization elements, helicases, and proteases 56, 57. These factors interact in a complex manner to control mitochondrial biosynthesis, differentiation, and functional development, thereby influencing the activation of mitochondrial pathways involved in programmed cell death. Mitochondrial biosynthesis is also sensitive to the cellular environment, signal transduction, and external stimuli 58. Malfunctions in this process can lead to nutrient and energy deficiencies within cells, impairing their normal function. Our study confirms that BYHW modulates the programmed cell death network by addressing mitochondrial biosynthesis dysfunction, providing experimental evidence for the MAPKK4-mediated multi-pathway death program in coronary microvascular endothelial cells. However, the *in vivo* regulatory mechanism of BYHW on p38 has yet to be fully elucidated.

### Buyang Huanwu decoction improves coronary microvascular injury after IR by inhibiting P38 mediated inflammatory storm

**Figure 6:**
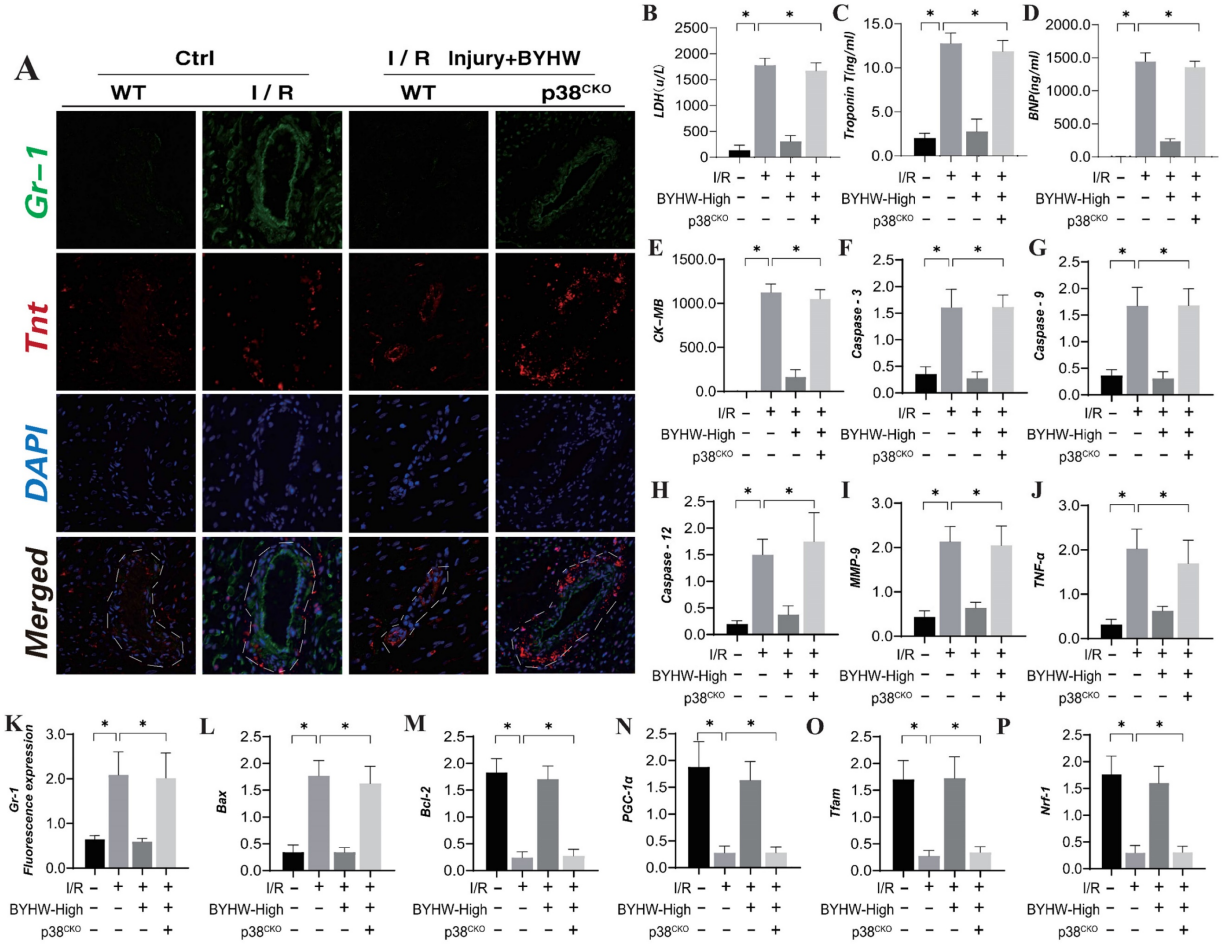
** Buyang Huanwu decoction improves coronary microvascular injury after IR by inhibiting P38 mediated inflammatory storm. (A, K)** Tissue immunofluorescence detection of Gr-1/Tnt expression levels; **(B)** LDH expression level; **(C)** Troponon T expression level; **(D)** BNP expression level; **(E)** CK-MB expression level; **(F)** Caspase-3 transcription level; **(G)** Caspase-9 transcription level; **(H)** Caspase-12 transcription level; **(I)** MMP-9 expression level; **(J)** TNF - α expression level; (L) Bax expression level; **(M)** Bcl-2 expression level; **(N)** PGC1- α transcription level; **(O)** Tfam transcription level; **(P)** Nrf-1 transcription level; Data are shown as mean ± SEM (n= ten independent cell isolations per group). *p<0.05.

The p38 mitogen-activated protein kinase (MAPK) is activated by various extracellular stresses and cytokines, and substantial evidence indicates that p38 MAPK is a key mediator of inflammatory responses and endothelial injury 59. To elucidate the role of the MAPKK4 downstream protein p38 in inflammation-driven coronary microvascular injury, we established a transgenic mouse model expressing p38 in microvascular endothelial cells following ischemia-reperfusion (IR) injury (P38^TG^). The experimental results demonstrated that p38 transgenic treatment negated the therapeutic effects of Buyang Huanwu decoction (BYHW) on microvascular injury. Furthermore, BYHW intervention significantly attenuated myocardial inflammation following IR, inhibited endothelial cell apoptosis, and enhanced mitochondrial biosynthesis (Figure 6A-P). However, the transgenic overexpression of p38 also abolished these beneficial effects of BYHW.

These findings suggest that p38 activation may impede the targeted therapeutic effects of BYHW on cell apoptosis and the inflammatory response. BYHW likely exerts its protective effects on endothelial cells by modulating apoptosis and inflammatory damage through the p38 pathway, thereby enhancing endothelial cell homeostasis and maintaining vascular endothelial stability. This conclusion is consistent with earlier animal studies involving MAPKK4, further supporting the notion that BYHW regulates cell apoptosis and improves coronary microvascular endothelial function via the MAPKK4-p38 axis.

## Discussion

Coronary microvascular injury after myocardial infarction has always been a difficult problem of concern in the medical community 60, 61. Under stress, acute occlusion of coronary arteries can further lead to pathological changes in endothelial cells caused by acute hypoxia/ischemia stimulation and intrinsic shear stress 62. A series of biochemical and metabolic stress changes occur in endothelial cells and cardiomyocytes, such as a shift in metabolic mode from aerobic glycolysis to anaerobic glycolysis, which leads to disturbances in intracellular acid-base balance and calcium homeostasis 63. In addition, oxidative stress mediated by ROS and increased production and secretion of inflammatory mediators mediated by MMP-9/IL-17 can further activate the Caspase mediated apoptotic pathway 64. If the duration of ischemia/hypoxia is prolonged (more than 20 minutes), myocardial cell apoptosis begins from the endocardium and expands towards the epicardium. Although endothelial cells are more tolerant to long-term ischemia than cardiomyocytes, myocardial injury and excessive activation of apoptotic genes after ischemia-reperfusion injury also led to swelling and rupture of microvascular endothelial cells, disruption of cell structural integrity, and changes in microvascular permeability, further resulting in loss of microvascular dilation response, hemodynamic disorders, and contraction of peripheral cells in the heart 65, 66. More importantly, microvascular injury in the ischemic area of the heart is mainly determined by the duration of myocardial ischemia, but the degree of myocardial ischemic injury may also be an important determining factor 67, 68. Therefore, synchronous treatment targeting myocardial ischemic injury and microvascular dysfunction is a widely concerned scientific issue in the medical community. In this study, the experimental results suggest that BY can dose-dependently improve ischemia-reperfusion injury after IR, and further inhibit microvascular inflammation storm, oxidative stress damage, and microvascular endothelial cell apoptosis. The P38/MAPKK4 gene modification experiment confirmed that the mechanism of BY on post-IR coronary microvascular injury may be achieved through the MAPKK4 pathway. And it also suggests that MAPKK4 can regulate the activity of downstream P38, thereby affecting coronary microvascular injury and mitochondrial pathway cell apoptosis after IR. BY may regulate the activity of P38 through MAPKK4 to produce a protective effect against microvascular injury after IR. Animal and cell experiments have also confirmed that the transgenic (overexpression treatment) of P38/MAPKK4 blocks the protective effect of BY on microvascular inflammation/oxidative stress damage. In the early stage, we have confirmed through experimental research that the effective active ingredients in BY have an improving effect on myocardial injury 28, 37. Quercetin alleviates myocardial ischemia-reperfusion injury caused by mitochondrial oxidative stress through DNA PKcs, regulates mitochondrial autophagy and mitochondrial dynamics, and maintains normal levels of mitochondrial energy metabolism 37. And it was found that quercetin can regulate the stability of DNA PKcs through SIRT5, maintain the synergistic effect of "mitochondrial autophagy unfolded protein response," and inhibit the activation of mitochondrial pathway cell necrotic apoptosis 37. The active ingredient quercetin in BY can regulate mitochondrial calcium homeostasis and mitochondrial quality control system abnormalities through the TMBIM6-VDAC1 interaction mechanism, which is helpful for the treatment of ischemic myocardial injury 28.

Coronary microvascular injury following myocardial infarction remains a significant challenge in the medical community 3, 69. Under stress conditions, acute occlusion of coronary arteries can precipitate pathological changes in endothelial cells, triggered by acute hypoxia/ischemia and intrinsic shear stress 70, 71. This leads to a cascade of biochemical and metabolic stress responses in endothelial cells and cardiomyocytes, including a metabolic shift from aerobic to anaerobic glycolysis, resulting in disturbances in intracellular acid-base balance and calcium homeostasis 72. Additionally, oxidative stress mediated by reactive oxygen species (ROS) and the increased production and secretion of inflammatory mediators, such as MMP-9 and IL-17, can further activate the Caspase-mediated apoptotic pathway 73. Prolonged ischemia/hypoxia (exceeding 20 minutes) initiates myocardial cell apoptosis starting from the endocardium and progressing towards the epicardium 74. Although endothelial cells exhibit greater tolerance to prolonged ischemia compared to cardiomyocytes, myocardial injury and the excessive activation of apoptotic genes following ischemia-reperfusion (IR) injury can lead to swelling and rupture of microvascular endothelial cells 45, 75. This results in the disruption of cell structural integrity, alterations in microvascular permeability, loss of microvascular dilation response, hemodynamic disorders, and contraction of surrounding cardiac cells. Importantly, the extent of microvascular injury in the ischemic heart region is primarily determined by the duration of myocardial ischemia, though the severity of myocardial ischemic injury is also a crucial factor. Consequently, the synchronous treatment of myocardial ischemic injury and microvascular dysfunction remains a critical area of focus in medical research.

In this study, our experimental results suggest that Buyang Huanwu decoction (BYHW) can dose-dependently improve IR-induced injury, while inhibiting microvascular inflammation, oxidative stress, and endothelial cell apoptosis. The P38/MAPKK4 gene modification experiments confirmed that the therapeutic effects of BYHW on post-IR coronary microvascular injury are likely mediated through the MAPKK4 pathway. This also indicates that MAPKK4 regulates downstream P38 activity, influencing coronary microvascular injury and apoptosis via the mitochondrial pathway following IR. BYHW may exert its protective effects against microvascular injury after IR by modulating P38 activity through MAPKK4. Both animal and cell experiments further confirmed that transgenic overexpression of P38/MAPKK4 negates the protective effects of BYHW on microvascular inflammation and oxidative stress. Previous studies have demonstrated that the active ingredients in BYHW significantly improve myocardial injury. Specifically, quercetin has been shown to alleviate myocardial ischemia-reperfusion injury by mitigating mitochondrial oxidative stress via DNA-PKcs, regulating mitochondrial autophagy and dynamics, and maintaining normal mitochondrial energy metabolism. Moreover, quercetin has been found to stabilize DNA-PKcs through SIRT5, ensuring the synergistic function of the "mitochondrial autophagy unfolded protein response," and inhibiting mitochondrial pathway-mediated necrotic apoptosis 37. Additionally, quercetin in BYHW regulates mitochondrial calcium homeostasis and corrects mitochondrial quality control system abnormalities through the TMBIM6-VDAC1 interaction mechanism, which is beneficial for treating ischemic myocardial injury 28.

While myocardial reperfusion is essential for maximizing myocardial recovery, it also exacerbates damage to the myocardium and its microvascular system. Historically, the focus has predominantly been on myocardial injury, often neglecting microvascular injury 76. This dichotomy, resulting from myocardial ischemia-reperfusion, is a complex process that intensifies microvascular injury initially caused by ischemia. Research indicates that reperfusion injury accounts for 50% of the total irreversible myocardial damage and is a significant factor contributing to severe microcirculatory dysfunction. Our preliminary studies revealed that myocardial reperfusion not only induces intracellular calcium overload but also triggers VDAC1-mediated opening of the mitochondrial permeability transition pore (mPTP) and the activation of inflammatory cascades 77. However, the active ingredients in Buyang Huanwu decoction (BYHW) have been shown to effectively mitigate myocarditis injury via the TMBIM6-VDAC1 pathway, consistent with our findings. This suggests that BYHW is a potential therapeutic agent for treating microvascular dysfunction following ischemia-reperfusion injury, with its targeted regulatory effects closely tied to the apoptosis of coronary microvascular endothelial cells.

Cell apoptosis is intricately regulated by genes that either inhibit (e.g., CrmA, Mcl-1, Bcl-2, Bcl-xL, Bcl-w) or promote (e.g., p53, Myc, Fas, Bax, Bad, Bak) apoptosis, as well as by bidirectionally regulated genes (e.g., Bcl-x, C-Myc) 43, 44, 78. Various inducing factors lead to different apoptotic pathways: internal pathways regulated by Bcl-2 family proteins, external pathways triggered by caspase activation mediated by tumor necrosis factor, and pathways mediated by endoplasmic reticulum stress-related factors 47, 79. Under stress conditions such as abnormal sympathetic activity, disrupted cardiac electrical activity, myocardial injury, vascular endothelial injury, and energy metabolism dysfunction, the apoptosis of myocardial and vascular endothelial cells can be accelerated^80^, ^81^. Additionally, factors such as inflammation, high glucose, high-fat environments, endotoxins, free radicals, oxidatively modified low-density lipoprotein (oxLDL), and aberrant platelet activation can also induce apoptosis in vascular endothelial cells. Previous studies have demonstrated that quercetin, an active ingredient in BYHW, ameliorates myocardial cell apoptosis following ischemia-reperfusion injury via the DNA-PKcs-SIRT5 pathway. Quercetin further regulates calcium homeostasis and mPTP dysfunction through the TMBIM6-VDAC1 interaction, thereby mitigating necrotic apoptosis via the mitochondrial pathway. Additionally, quercetin improves myocardial injury and cell apoptosis post-myocardial infarction by modulating the "mitochondrial autophagy unfolded protein response" through the SIRT5-β-tubulin axis. This study further elucidates the anti-apoptotic mechanism of BYHW targeting coronary microvascular endothelial cells and confirms its regulatory effect on inflammation and oxidative stress via the MAPKK4-p38 interaction mechanism at the molecular level.

Mitogen-activated protein kinases (MAPKs) are serine/threonine protein kinases widely present in eukaryotic cells, responsible for transmitting various extracellular stimuli to intracellular signaling pathways and mediating diverse cellular responses 82. The MAPK family of signaling pathways identified in current research primarily includes extracellular signal-regulated kinase 1/2 (ERK1/2), p38 mitogen-activated protein kinase (p38 MAPK), c-Jun N-terminal kinase (JNK), and extracellular signal-regulated kinase 5 (ERK5). Although each MAPK pathway has distinct characteristics, they share common mechanisms 83. All MAPK pathways transmit extracellular signals to cells via a cascade of tertiary kinases, regulating various physiological and pathological processes, including growth, differentiation, and apoptosis of myocardial and endothelial cells 84. Among these, the p38 pathway is the most extensively studied. It can be activated by various stimuli, has relatively independent functions, and exhibits broad interactions with other MAPK pathways, with either synergistic or inhibitory effects. Previous studies have shown that H_2_O_2_-induced oxidative stress significantly activates cAMP response element-binding protein (CREB), enhances transcription and expression of IP3R and VDAC, and activates mitochondrial-dependent death pathways. Melatonin can protect cardiac microvascular endothelial cells (CMECs) from oxidative stress by stimulating the MAPK/ERK pathway 85, 86. This study confirms that MAPKK4 mediates energy metabolism dysfunction in endothelial cells and the activation of apoptosis-regulatory genes in the mitochondrial pathway after ischemia-reperfusion (IR) by regulating p38 activity. *In vitro* and *in vivo* experiments further confirm that Buyang Huanwu decoction (BYHW) ameliorates coronary microvascular injury through the MAPKK4-p38 axis. These findings provide new avenues for potential drug development and clinical translation in treating coronary microvascular injury.

BYHW is a well-established traditional formula for the clinical treatment of cardiovascular and cerebrovascular diseases, with significant therapeutic effects demonstrated over many years. Previous studies have shown that this formula can reduce inflammatory responses and exert therapeutic effects on chronic cerebral ischemic injury by targeting AKT1, ALB, TP53, IL-1β, and IL-6^14^. Further research indicates that BYHW can improve ischemic injury in the brain after IR by targeting Aprt, Pde1b, Gpd1 and HEXB, as well as pathways involved in glycerophospholipid metabolism 87. Our research aligns with these previous findings, further confirming BYHW's mechanisms of action on microcirculatory disorders and identifying relevant therapeutic targets for this condition. Specifically, our study confirms the pathway through which BYHW improves microvascular injury after myocardial infarction by regulating p38 via MAPKK4, offering a preliminary basis and reference for MAPK-targeted drug therapy research.

Despite the comprehensive exploration and confirmation of BYHW's targeted therapeutic mechanisms, several issues remain unresolved in our *in vitro* and *in vivo* experiments. Firstly, we did not explore the impact of subcellular phenotypes, such as mitochondria or the endoplasmic reticulum, on the apoptosis of coronary microvascular endothelial cells. Future research will focus on confirming BYHW's targeted regulatory effects on organelles through *in vitro* and subcellular interaction experiments to elucidate its upstream regulatory influence on apoptosis. Secondly, we have not yet validated the effects of other endothelial cell programmed cell death pathways, such as necroptosis, pyroptosis, and ferroptosis, on the pathological mechanisms of post-IR coronary microvascular injury and BYHW's treatment mechanisms. Future experiments will investigate BYHW's targeted effects on various programmed cell death regulatory mechanisms in endothelial cells. Finally, we have not validated the interaction mechanisms between MAPKK4 and p38, nor the effects of BYHW on cell apoptosis, mitochondrial respiratory chain complexes, or mitochondrial biosynthesis, through immunoprecipitation and immunoblotting experiments. Future studies will aim to confirm these interactions through more advanced experimental methods.
